# Effects of Multi-Walled Carbon Nanotubes on Mechanical Properties and Microstructure of Ordinary Portland Cement–Sulfoaluminate Cement Repair Mortar

**DOI:** 10.3390/ma18163748

**Published:** 2025-08-11

**Authors:** Qun Zhou, Runzhuo Cao, Xiaodong Ma

**Affiliations:** 1 College of Urban Construction, Xi’an Siyuan University, Xi’an 710038, China; 2 College of Materials Science and Engineering, Xi’an University of Architecture & Technology, Xi’an 710055, China; maxiaod@xauat.edu.cn

**Keywords:** multi-walled carbon nanotubes, repair mortar, mechanical properties, microstructure

## Abstract

Multi-walled carbon nanotubes (MWCNTs) with high thermal conductivity and electrical conductivity are frequently considered as ideal nano-reinforced materials for the future. This paper investigated the potential application of MWCNTs in ordinary Portland cement–sulfoaluminate cement (OPC-SAC) repair mortar by analyzing mechanical and microstructural changes caused by MWCNTs. The test results revealed that MWCNTs greatly increased the strength of OPC-SAC binary repair mortar in the early days, and promoted sustained growth of long-term strength. The 10.39%/9.3 MPa increases in compressive strength can be attributed to 0.10 wt.% MWCNTs. MWCNTs promotes hydration of OPC-SAC composites through functional groups and nucleation effects, resulting in more C-S-H gels and AFt crystals. The X-ray computed tomography (X-CT), mercury intrusion porosimetry (MIP), and scanning electron microscope (SEM) results indicate that the nanofibers (MWCNTs) optimize the microstructure and microstructure of the composites. The nanofibers with high aspect ratio results enhance the crosslinking between hydration products, improve complexity (higher D_s_) and integrity (more crosslinking sites), and reduce the formation and propagation of microcracks through bridging. The filling effect of nanoparticles refines the pore and reduces the pore volume, especially the volume of medium capillary pores. It is precisely these combined actions that improve the engineering performance of OPC-SAC binary repair mortar.

## 1. Introduction

Concrete is a typical brittle material with low tensile strength and strain ability, which makes concrete structure prone to cracking and premature deterioration and damage, leading to a series of durability problems and incalculable maintenance costs [1,2,3]. Nanocracks are formed within the gel material, and then gradually expand into micro and macro cracks. In general, large fibers can only delay the propagation of cracks, but cannot prevent the formation of cracks. Nanosized fibers/tubes could theoretically prevent or delay crack initiation [4,5,6]. To overcome the shortcomings of traditional cement-based materials, multi-walled carbon nanotubes (MWCNTs) reinforcement has attracted extensive attention in industry and academia [7,8,9,10].

Carbon nanotubes (CNTs) are considered ideal nano-reinforced materials for high strength and aspect ratio, and excellent electrical conductivity and thermal conductivity [2,11,12]. CNTs have positive effects on the improvement of flexural/compressive strength, elastic modulus, and fracture energy of concrete. Li et al. [13] found that 0.5 wt.% of MWCNTs to cement-based materials increased the flexural and compressive strength by 25% and 19%, respectively. Mohsen et al. [14] confirmed that the optimal dosages of CNTs was 0.25% based on analysis of variance, and the flexural and compressive strength could be increased by 60% and 25%, respectively. Chen et al. [3] reported that adding 0.02 wt.% and 0.05 wt.% MWCNTs to composites can improve the fracture toughness by 9.08% and 14.06%, respectively, with ligament bridging being the main toughening mechanism. Multiple researchers showed that the CNTs can obviously decrease the resistivity of concretes, regardless of their water content [15,16,17,18]. Xu et al. [19] used nanoindentation to characterize the effect of CNT on cement hydration, and found that CNT promoted the formation of high-stiffness C-S-H gels, optimized nanostructures, and reduced nanopores. The enhancement mechanism of CNTs in cement composites includes filling micropores owing to their small dimensions, bridging narrow cracks through their high aspect ratio, and accelerating C-S-H growth through nucleation effects [20,21,22]. Uniformly dispersed CNTs can fill the void and connect the hydration products, improve the compactness and fracture energy of the composites, improve the interface bonding characteristics, and inhibit crack propagation.

Ordinary Portland cement–sulfoaluminate cement (OPC-SAC) composites have the characteristics of controllable setting and hardening rate, similar to the thermal expansion performance of ordinary concrete, high early strength, and can compensate for shrinkage, etc., which is suitable for repair projects with tight time window requirements, such as national defense engineering and emergency repair engineering [23,24,25,26]. However, it is difficult to achieve the requirement of early strength and continuous increase in late strength only by adjusting the mixing ratio of OPC and SAC, so it is necessary to introduce new components to achieve this technical requirement.

In view of this, the performance advantages of MWCNTs and OPC-SAC repair mortar can be combined to prepare repair materials with better performance. In addition, existing research mainly focuses on the impact of MWCNTs on ultra-high-performance concrete, while the effects on rapid repair materials, such as OPC-SAC composites, have not been reported. Hence, our main objective is to explore the influence of MWCNTs on the macroscopic properties and microstructure of OPC-SAC binary mortar as repair material. The strength of OPC-SAC with MWCNTs were measured, and the modification mechanisms of MWCNTs on the meso- and micro-structure of OPC-SAC composites was investigated in detail through X-ray diffraction (XRD), thermogravimetric (TG), scanning electron microscope (SEM), X-ray computed tomography (X-CT), and mercury intrusion porosimetry (MIP) analyses.

## 2. Experimental Schemes

### 2.1. Materials

The cementitious materials used to prepare the OPC-SAC repair mortar were sulfoaluminate cement (SAC) and ordinary Portland cement (OPC), and were provided by Yaobai Special Cement Group Co., Ltd. (Xi’an, China) and Tangshan Polar Bear Special Cement Co., Ltd. (Tangshan, China), respectively. Their chemical composition is demonstrated in Table 1. The MWCNTs were purchased from Shanghai Naiou Nanotechnology Co., Ltd. (Shanghai, China), and MWCNTs’ parameters are listed in Table 2. Cetyltrimethyl ammonium bromide (CTAB) was purchased as the surfactant for MWCNTs from Xilong Scientific Co., Ltd. (Shantou, China). The polycarboxylate superplasticizer with a reducing water rate of 30% was used to guarantee the workability of OPC-SAC repair mortar, which was provided by Sobute New Materials Co., Ltd. (Nanjing, China).

### 2.2. Sample Preparation

The dosages of MWCNTs were 0.05%, 0.075%, 0.10%, 0.125%, and 0.15% by mass of cement, respectively. A reference group was also prepared without MWCNTs. The mix proportions of MWCNTs modified composites were tabulated in Table 3. Mix code represents the dosage of MWCNTs. For example, MT-0.10 represents the addition of MWCNTs with a mass fraction of 0.1% into OPC-SAC composites. In this research, the mass ratio of CTAB to MWCNTs was fixed at 0.4.

**Figure 1:**
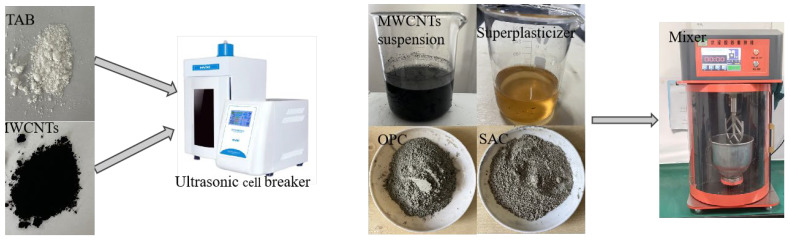
Main flowchart for the preparation of repair mortar.

The main flowcharts for the preparation of repair mortar are shown in Figure 1. The fresh mixtures were cast into steel molds with the size of (40 × 40 × 160 mm^3^), and then vibrated for 2 min. All mortars were demolded after 24 h and then cured in standard curing boxing with temperature of 20 ± 1 °C and relative humidity 90 ± 5% to design age. Furthermore, MWCNTs paste was also prepared for analyses of chemical composition and microstructure.

### 2.3. Dispersion of MWCNTs

The dispersion of nanomaterials is always a difficult problem for nano-engineered concrete composites [10,27]. Currently, CNTs suspensions can be prepared through physical and chemical methods [18,28]. A MWCNT suspension was prepared by surfactant modification and ultrasonic dispersion in this study. First, we added the CTAB to the water and stirred at 200 R/min to dissolve it completely. Secondly, MWCNTs were added to a solution containing surfactants, and dispersed by ultrasound at 20 kHz for 30 min. Finally, the MWCNTs suspension after ultrasonic dispersion was allowed to stand for 24 h to eliminate any bubbles introduced during the dispersion process.

### 2.4. Test Programs

The strength of OPC-SAC repair mortars were tested based on Chinese standard GB/T 17671-2021 [29]. Three mortars were subjected to three-point bending at a loading rate of (50 ± 10) N/s. At a loading rate of (2400 ± 200) N/s, the compressive strength of six prisms made by three-point bending was tested.

Mineral phases composition analysis was achieved using the XRD and TG analysis. Both XRD and TG samples were mechanically ground and then passed through an 80 μm sieve. The XRD tests were performed using a D/MAX-3C X-ray diffractometer (Bruker AXS GMBH, Karlsruhe, Germany) at a 2/min scan speed were employed over 5° to 60° 2θ. In nitrogen atmosphere, TG tests were performed from 25 to 900 °C, and with a 10 °C/min heating rate. The microstructures of the paste specimens modified with MWCNTs were investigated by SEM (Model Gemini SEM500, Carl Zeiss (Shanghai) Management Co., Ltd., Shanghai, China). Characterization of the pore structure of OPC-SAC mortars using MIP and X-CT. MIP testing using Model AutoPore IV 9500 (Micromeritics instrument (Shanghai) Ltd., Shanghai, China) automatic mercury porosimeter. The X-CT experiment used the Zeiss METROTOM-800 CT industrial machine (Carl Zeiss (Shanghai) Management Co., Ltd., Shanghai, China) to obtain 700 two-dimensional slice images, with corresponding image pixels of 26 µm. Both SEM and MIP samples sizes are approximately 5 mm × 5 mm × 5 mm. Before performing XRD, TG, SEM, MIP, and X-CT tests, all samples need to undergo vacuum drying pre-treatment.

The complexity of pore spatial distribution form can be estimated by surface fractal dimension (D_s_), this model has been proved to be accurate in describing D_s_. D_s_ can be calculated using the following formula:

ln(W_n_/r_n_^2^) = D_s_ ln(V_n_^1/3^/r_n_) + C (1)

where W_n_ stands for cumulative surface energy, V_n_ represents the volume of mercury injection into the pores during the nth period, r_n_ denotes the pore radius, subscript n is mercury intrusion stage, and C is a constant.

## 3. Results and Discussion

### 3.1. Mechanical Properties

**Figure 2:**
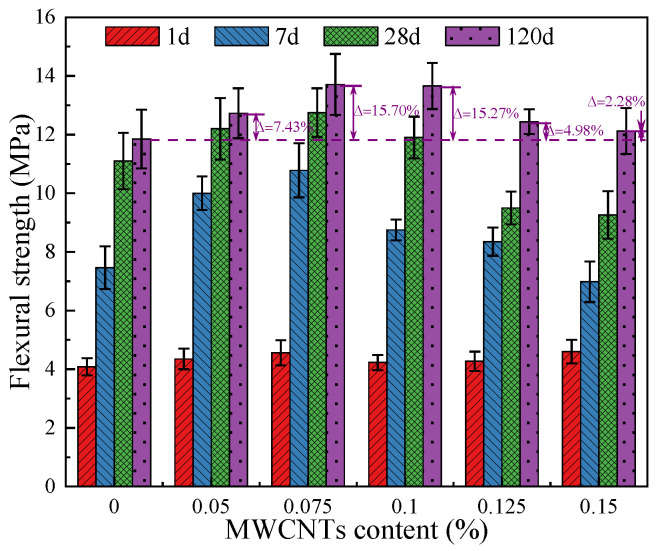
Flexural strength of mortars at different age.

Figure 2 presented the flexural strength of OPC-SAC composites with different MWCNT contents. An appropriate amount of MWCNTs is beneficial to increase the flexural strength of mortar. For example, compared to MT-0, the flexural strength of MT-0.05, MT-0.075, MT-0.10, MT-0.125, and MT-0.15 increased by 7.43%, 15.70%, 15.27%, 4.98%, and 2.28%, respectively. The flexural strengths of MT-0.10 standard cured for 1, 7, 28, and 120 d are 4.2, 8.8, 11.9, and 13.7 MPa, respectively, which is higher than that of MT-0 at the same age. The reduction of flexural strength may be due to the aggregation and entanglement of excess MWCNTs in mortar, resulting in the formation of local defects [28].

**Figure 3:**
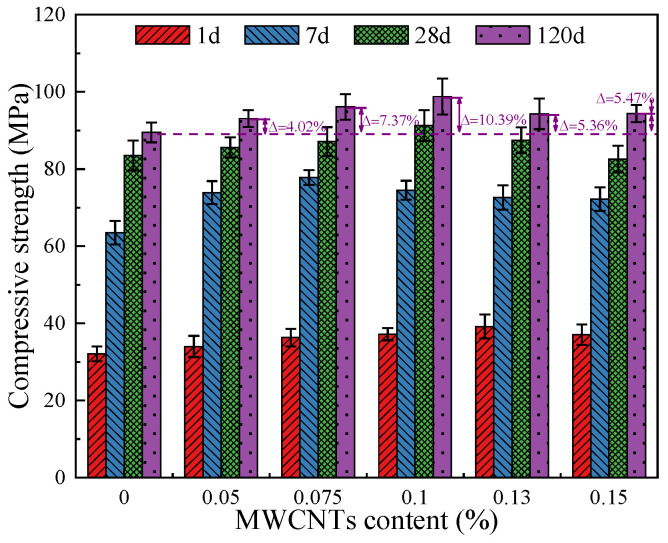
Compressive strength of mortars at different age.

Figure 3 exhibits the compressive strength of the MWCNTs modified OPC-SAC mortar and reference group at different curing ages (including 1, 7, 28 and 120 d), and the fitted curves between curing age and strength are also presented. In general, MWCNTs contributes to the strength growth of OPC-SAC composites. Specifically, the strength first increases and then decreases with the increase in MWCNTs dosage. The compressive strength of MWCNTs modified OPC-SAC mortar was higher than that of MT-0 regardless of the ages. The compressive strength of MT-0.10 was the highest, which was 91.3 and 98.8 MPa at 28 and 120 d, 9.3% and 10.4% higher than that of MT-0, respectively. The reasons why MWCNTs promote strength growth can be summarized as: (1) the adsorption and nucleation effects of MWCNTs particles are beneficial for the hydration of the matrix, thus improving its compactness; (2) MWCNTs inhibited the formation and propagation of microcracks by bridging hydration products; (3) fills the pores of matrix through the micro-aggregate effect [2,18,19,30,31,32,33,34,35].

### 3.2. X-Ray Diffraction

**Figure 4:**
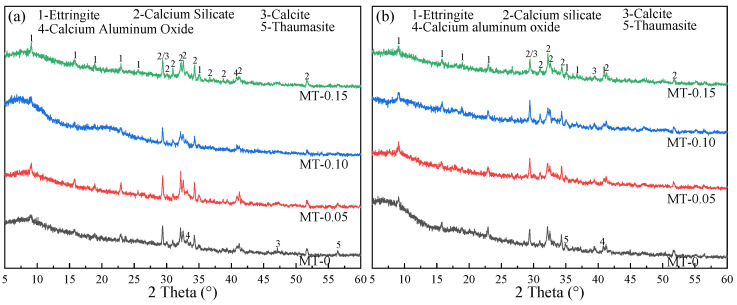
XRD spectra of pastes with MWCNTs at different cure age: (**a**) 7 d; (**b**) 120 d.

Figure 4 shows XRD patterns of MT-0, MT-0.10, and MT-0.15 at 7 and 120 d. From Figure 4a, the peak positions of ettringite, calcium silicate, calcium aluminum oxide, and calcite in MT-0, MT-0.10, and MT-0.15 are basically the same. After 120 d of curing, the peak intensity of C_3_S and C_2_S was lower than that after curing for 7 d, indicating that more C-S-H was generated, which was in line with the hydration mechanism [36]. Notably, unhydrated clinkers are still present, even at 120 d of standard curing, which accounts for the continuous improvement in OPC-SAC composites performance. The diffraction peak intensity corresponding to clinker (C_2_S and C_3_S) in MWCNTs modified paste (MT-0.05, MT-0.10, and MT-0.15) was relatively lower in comparison to those of the reference group. This means that the hydration degree of the MWCNTs modified paste was relatively higher than that of MT-0. The reasons can be concluded as follows: on the one hand, MWCNTs adsorb a certain amount of calcium ions through adsorption effect, promoting AFt formation; on the other hand, MWCNTs with high length–diameter ratios can be used as nucleating agents for C-S-H gels [37].

### 3.3. TG Analysis

**Figure 5:**
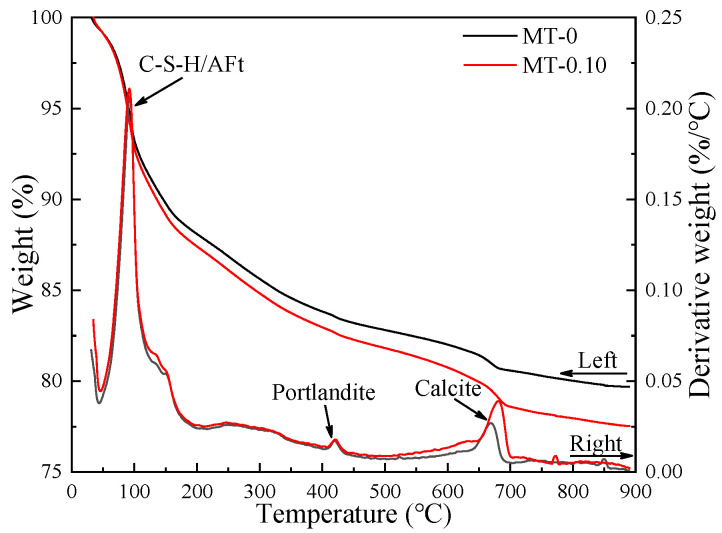
TG analysis results of pastes at 120 d.

Figure 5 presents TG curves of MT-0 and MT-0.10 for 120 d. On the curve, three weight loss peaks can be observed, corresponding from left to right to the dehydration of ettringite and C-S-H gel, the dehydration of portlandite, and the decomposition of calcite [38,39]. The content of chemically bound-water (CBW) was determined on the basis of previous relevant studies [40]. The TML of MT-0.10 is 22.47%, higher than the 20.31% of MT-0, which decreased by 9.61%. The CBW of MT-0.10 is 18.30%, higher than that of MT-0 (16.05%). The hydration degree of MT-0.10 was higher, illustrating that the MWCNTs promotes the hydration of the binder, corresponding to strength (Figure 3) and XRD results (Figure 4). Previous studies have also yielded similar findings, suggesting that MWCNTs can serve as nucleation agents to speed up the hydration process [2,41,42].

### 3.4. X-CT Analysis

**Figure 6:**
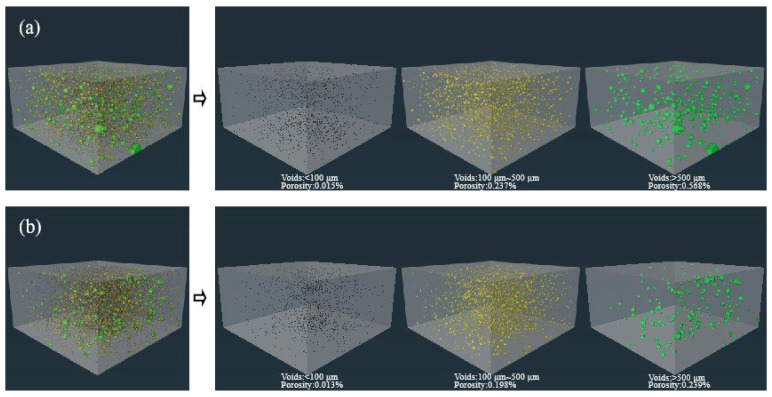
Three-dimensional pore reconstruction image of mortar: (**a**) MT-0; (**b**) MT-0.10.

To truly reflect the original structure of the internal pores of the sample, X-CT scanning was performed on the mortar, and three-dimensional reconstruction was performed using AVIZO 2020.1 software to statistically analyze the pore information. After standard curing for 120 d, the three-dimensional pore reconstruction images of MT-0 and MT-0.10 are shown in Figure 6, and the corresponding pore characteristic parameters are shown in Table 4. The black, yellow, and green colors in the figure represent pores with equivalent diameters of <100 μm, 100~500 μm, and ≥500 μm, respectively. From the reconstructed image, it can be intuitively seen that compared with MT-0, the pore size in MT-0.10 is smaller, the number is fewer, the pore size is more concentrated, and the degree of isolation between each other is higher. Therefore, the three-dimensional structure visualization image can intuitively show that the defect size in MT-0.10 is smaller, the number is fewer, and the structure is denser.

Quantitative calculation shows that in MT-0, the micrometer porosity of equivalent diameter < 100 μm, 100 μm~500 μm, and ≥500 μm is 0.015%, 0.237% and 0.568%, respectively. The overall porosity and average equivalent diameter are 0.820% and 86.1 μm, respectively. In MT-0.10, compared with MT-0, the micrometer porosity of equivalent diameter < 100 μm, 100~500 μm, and ≥500 μm decreased by 13.3%, 16.5%, and 57.9%, respectively, and the pore volume at all levels decreased to different degrees. The overall micrometer porosity and average equivalent diameter were reduced to 0.450% and 70.3 μm.

It can be seen that the quantitative analysis results were consistent with the qualitative observation trend. The addition of MWCNTs appropriately divided large pores into small and medium-sized pores, significantly improving the degree of compactness and refining the pore structure.

### 3.5. MIP Analysis

**Figure 7:**
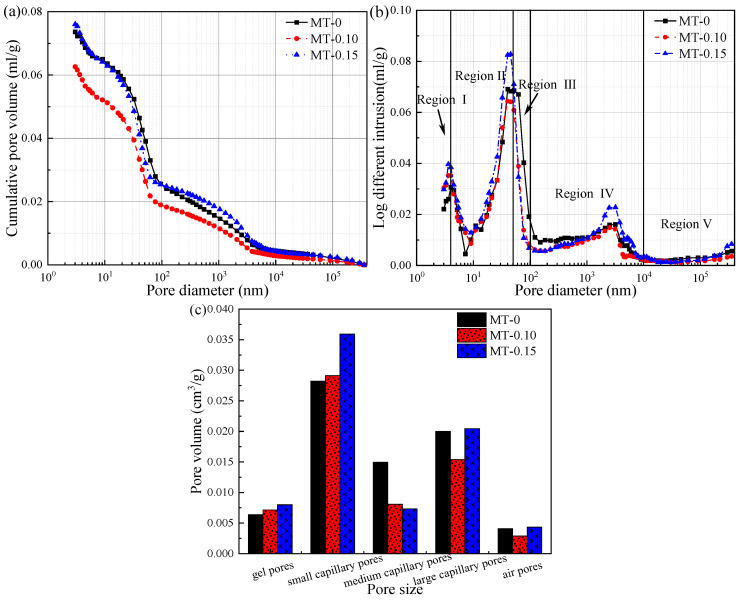
MIP results of mortars containing MWCNTs:. (**a**) Cumulative pore volume; (**b**) Pore size distribution; (**c**) Specific pore volume.

Since X-CT can only detect air pores, and cannot observe gel pores, the nano-scale pores in the mortar are quantitatively analyzed by MIP test. The MIP results of mortar samples with MWCNTs are shown in Figure 7.

When the dosage of MWCNTs gradually increased to 0.15%, the cumulative pore volume of mortar samples first decreased and then increased. Specifically, compared to MT-0, the cumulative pore volume of MT-0.10 decreased by 15.01%, while the that of MT-0.15 slightly increased by 3.17%. Furthermore, the cumulative pore volume curve of MT-0.10 moves downward as a whole, indicating that the pore structure has been refined. Based on the studies on pore structure, regions I, II, III, IV, and V, respectively, represent gel pores, small capillary pores, medium capillary pores, large capillary pores, and air pores, and the corresponding pore diameters are <5 nm, 5–50 nm, 50–100 nm, 100–10,000 nm, and ˃10,000 nm [35,43]. From Figure 7b, the MWCNTs did not significantly change the pore distribution of OPC-SAC mortar.

To quantitatively analyze the impact of MWCNTs on pore structure, the pore volumes in five regions were calculated based on the cumulative pore volume in Figure 7b, as shown in Figure 7c. Contrary to MT-0, the gel and small capillary pore volumes of MT-0.10 increased by 11.89% and 3.15%, respectively, while the medium capillary, large capillary and air pore volumes decreased by 45.96%, 23.05%, and 30.00%, respectively. The gel, small capillary, and medium capillary pore volumes of MT-0.15 showed the same trend as that of MT-0.10, but the change trend of large capillary and air pore was opposite. The results revealed that the medium capillary, large capillary, and air pores were transformed into gel pores and small capillary pores by appropriate MWCNTs, which greatly increased the volume of regions I and II.

Table 5 presents the pore characteristic parameters of MWCNTs modified OPC-SAC and the control group. The porosity change rule of mortars is similar to cumulative pore volume. MWCNTs lowers the medium pore diameter and most probable diameter of composites. To summarize, MWCNTs can induce the pore refinement, which is likely to convert harmful pores, such as air and large capillary pores, into less harmful pores, such as gel pores and small capillary pores, enhance matrix densification, and thus promote the sustained growth of the strength of OPC-SAC repair mortar.

**Figure 8:**
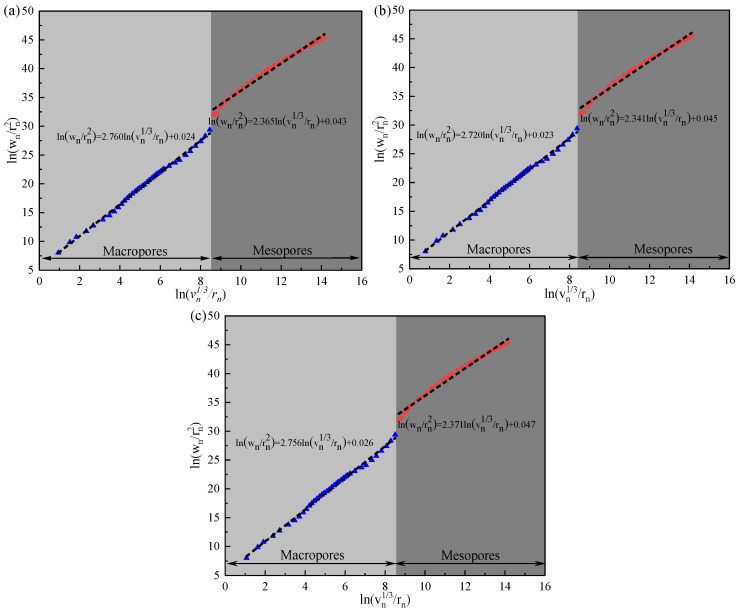
Relationship between ln(v_n_^1/3^/r_n_) and ln(w_n_/r_n_^2^): (**a**) MT-0; (**b**) MT-0.10; (**c**) MT-0.15.

According to the MIP results and Equation (1), the relationship between ln(v_n_^1/3^/r_n_) and ln(w_n_/r_n_^2^) is shown in Figure 8, and the corresponding D_s_ and correlation coefficient are shown in Table 6. The regions represented by macropores and mesopores are related to the accumulation pattern formed by hydration reactions and the microstructure of C-S-H [44,45,46]. High D_s_ denotes a complex spatial distribution and strong filling ability of pores [46,47]. The D_s_ values were between 2.341 and 2.760, and the related coefficients were higher than 0.95, indicating that the fitting has a good linear relationship; that is, the pore structure has obvious multifractal characteristics. In Figure 8, D_s_ of mesopores for MT-0, MT-0.10, and MT-0.15 are 2.365, 2.341, and 2.371, respectively, and that of macropores are 2.760, 2.720, and 2.756, respectively. The decrease in the D_s_ value of MT-0.10 may be due to the decrease in the porosity of the composites, while the change of the D_s_ value of MT-0.15 indicates that excess MWCNTs increases the heterogeneity of C-S-H, but weakens the filling pattern between particles.

### 3.6. SEM Analysis

**Figure 9:**
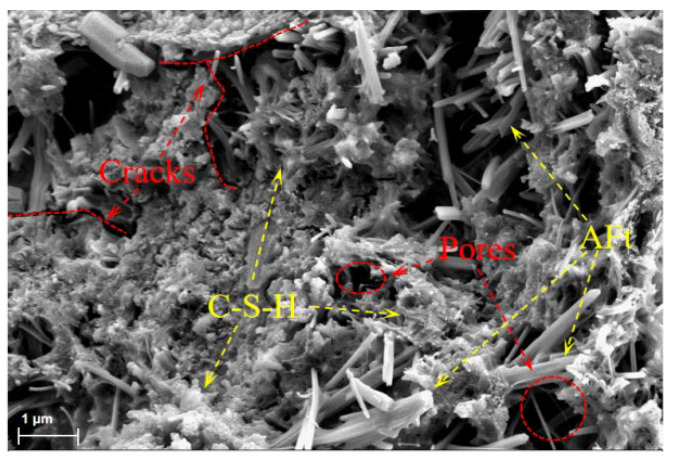
SEM image of MT-0 at 120 d.

**Figure 10:**
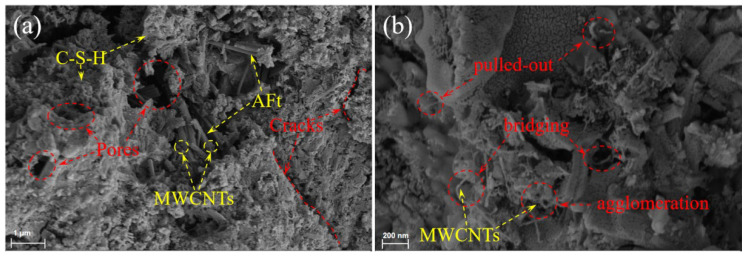
SEM images of MT-0.10 at 120 d: (**a**) ×10,000; (**b**) ×40,000.

To further explore the relationship between microstructures and mechanical properties, we observed the microstructural morphology of the sample, as shown in Figure 9 and Figure 10. Non-uniform microstructures composed of capillary pores, air pores, cracks, C-S-H gels, and AFt was observed in all samples. As depicted in Figure 10, MWCNT nanofibers randomly distributed in the OPC-SAC matrix effectively connect the hydration products to the unhydrated clinker, improving the strength and toughness of the OPC-SAC mortars. Furthermore, MWCNTs also acted as fiber bridging, restraining the initiation and propagation of cracks until the MWCNTs pulls out or breaks. However, owing to van der Waals forces, MWCNTs will partially agglomerate and intertwine, forming local defects in the matrix, resulting in weakened binding between the MWCNTs and the hydration products or leaving pores.

## 4. Conclusions

The influence of MWCNTs on the properties of OPC-SAC mortar were analyzed, and the modification mechanism was discussed through mineralogy and microstructure analysis. The chief findings have been summarized as follows:

1. Incorporation of MWCNTs improves the mechanical strength of OPC-SAC mortar. Contrary to that of OPC-SAC mortar without MWCNTs, the compressive strength of OPC-SAC mortar with 0.10 wt.% MWCNTs increased by 10.39%/9.3 MPa at most. Meanwhile, the flexural strength can be increased by 15.70%/1.9 MPa by adding 0.075 wt.% MWCNTs.
2. Dispersed MWCNTs promote hydration of OPC-SAC composites (especially in the early stages) through functional group and nucleation effects, but did not promote the formation of new substances.
3. The X-CT, MIP, and SEM results indicate that the MWCNTs optimizes the microstructure and microstructure of the composites. One role is to cause partial cross-linking occurs between hydration products, which enhances the complexity (higher D_s_) and integrity of the binder (more cross-linking sites). The other one is to reduce the generation and propagation of microcracks through nanofiber bridging. The third is to optimize the pore structure and reduce the porosity, especially the volume of medium capillary pores.

## Figures and Tables

**Table 1:** Chemical composition of raw materials.

Mass Fraction (w/%)	CaO	Al_2_O_3_	SiO_2_	Fe_2_O_3_	MgO	SO_3_	K_2_O	Na_2_O	LOI
OPC	68.62	3.42	17.52	4.61	1.79	1.23	1.10	0.66	1.05
SAC	42.70	36.27	6.56	2.48	1.55	8.75	0.17	0.22	0.70

**Table 2:** Parameters of the MWCNTs.

Out Diameter (nm)	Inner Diameter (nm)	Length (um)	Specific Surface Area (m^2^/g)	Purity (%)
10–20	5–10	20–30	˃250	˃95

**Table 3:** Mix proportions of mortars (g).

Sample Code	MWCNTs	OPC	SAC	Sand	Water	Superplasticizer
MT-0	0	800.0	200.0	800	250	6
MT-0.05	0.50	799.6	199.9	800	250	8
MT-0.075	0.75	799.4	199.9	800	250	8
MT-0.10	1.00	799.2	199.8	800	250	10
MT-0.125	1.25	799.0	199.8	800	250	10
MT-0.15	1.50	798.8	199.7	800	250	12

**Table 4:** Pore structure characteristic parameters of mortar derived from X-CT.

Simple Code	Porosity (%)	Equivalent Diameter (μm)	Pore Quantity
MT-0	0.82	86.1	27,042
MT-0.10	0.45	70.3	22,874

**Table 5:** Characteristic of pore structure of mortars.

Sample Code	Most Probable Aperture/nm	Average Pore Diameter/nm	Medium Pore Diameter/nm	Porosity/%
MT-0	40.3	22.5	55.6	15.17
MT-0.10	40.3	17.8	43.4	13.34
MT-0.15	45.3	19.0	43.9	15.86

**Table 6:** D_s_ of the pore surfaces.

Simple Code	Mesopores		Macropores	
	Fractal Dimension	Correlation	Fractal Dimension	Correlation
MT-0	2.365	0.990	2.760	0.997
MT-0.10	2.341	0.989	2.720	0.997
MT-0.15	2.371	0.988	2.756	0.997

## Data Availability

The original contributions presented in the study are included in the article, further inquiries can be directed to the corresponding authors.
